# Shoot-Root Communication Plays a Key Role in Physiological Alterations of Rice (*Oryza sativa*) Under Iron Deficiency

**DOI:** 10.3389/fpls.2018.00757

**Published:** 2018-06-05

**Authors:** Lin Chen, Gaopeng Wang, Pengfei Chen, Honglei Zhu, Shaohua Wang, Yanfeng Ding

**Affiliations:** ^1^College of Agriculture, Nanjing Agricultural University, Nanjing, China; ^2^Key Laboratory of Crop Physiology and Ecology in Southern China, Ministry of Agriculture, Nanjing Agricultural University, Nanjing, China; ^3^Jiangsu Collaborative Innovation Center for Modern Crop Production, Nanjing Agricultural University, Nanjing, China

**Keywords:** rice (*Oryza sativa*), Fe deficiency, shoot, root, response

## Abstract

Iron (Fe) is an essential mineral element required for plant growth, and when soil availability of Fe is low, plants show symptoms of severe deficiency. Under conditions of Fe deficiency, plants alter several processes to acquire Fe from soil. In this study, we used rice cultivars H 9405 with high Fe accumulation in seeds and Yang 6 with low Fe accumulation in seeds to study their physiological responses to different conditions of Fe availability. In both shoots and roots, the responses of ROS enzymes, leaf and root ultrastructure and photosynthetic system to iron deficiency in Yang 6 were much sensitive than those in H 9405. For the distribution of iron, the iron content was much higher in roots of Yang 6, in contrast to higher shoot content in H 9405. Differential responses were shown with the Fe content in roots and shoots, which were the opposite in the two varieties; thus, we proposed the existence of long-distance signals. Then split root and shoot removal experiments were used to demonstrate that a long-distance signal was involved in the iron-deficient rice plant, and the signal strength was highly correlated with the functional leaves.

## Introduction

Iron (Fe) is an important mineral nutrient that is vital for a variety of cellular and other physiological functions ranging from metabolism to growth and development, including chlorophyll synthesis, respiration, redox reactions, and electron transfer. Although Fe is one of the most abundant elements in the geosphere, Fe deficiency has become a common problem in plant production worldwide, many plants suffer from Fe deficiency (Schmidt, 2003). This deficiency is because most Fe in soil exists as Fe oxides, phosphate, or other insoluble compounds (Grusak et al., 1999), limiting the uptake and effectiveness.

Based on molecular biology studies, plants activate processes that are correlated with Fe absorption, transfer, and utilization in adapting to a low Fe environment. Under iron deficiency, plants adopt two Fe-absorption strategies, reduction (strategy I) and chelation (strategy II). Dicots and non-gramineous monocots use the reduction strategy composed of H^+^ release, Fe^3+^ to Fe^2+^ reduction and Fe^2+^ transport into roots by the Fe-regulated transporter 1 (IRT1) (Colangelo and Guerinot, 2004; Dinneny et al., 2008). Gramineous plants use chelation in which the phytosiderophores (PSs), such as MAs (mugineic acids), are released into the rhizosphere to directly chelate Fe^3+^ in rice, the enzymes involved in the synthesis of MAs contain nicotianamine synthase (OsNAS1, OsNAS2); then, the resulting Fe(III)-PS complexes are absorbed by root cells via proteins in the YS family, such as yellow stripe 1 (YS1) in maize (Curie et al., 2001, 2009). In rice, OsYSL15 is the protein that transports Fe(III)-PS into roots. *OsYSL15* is up regulated under Fe deficiency and is expressed in the root epidermis (Inoue et al., 2009; Lee et al., 2009). Once Fe enters the root, it will be loaded into xylem and phloem to transport in shoot in complex forms. The YELLOW STRIPE 1-like 2 (OsYSL2) transporter is responsible for Fe(II)-NA transport across the plasma membrane and plays a pivotal role in phloem-mediated iron distribution (Koike et al., 2004).

A signal is likely required to transmit nutrient status from shoots to roots for the absorption of a variety of nutrients (Forde, 2002). For iron, indirect evidence indicates the existence of a long-distance signal. Vert et al. (2003) proposed a promotive model and a repressive model concerning the mechanism of the signal of iron uptake in *Arabidopsis thaliana*. In the promotive model, a signal generated in leaves is sent to roots and induces the expression of iron uptake genes under the iron-deficient condition; however, the signal is not sent to roots when iron is sufficient. In the repressive model, iron-sufficient shoots constitutively express a signal, which suppresses the root iron uptake response; the signal disappears under the iron-deficient condition.

Several hormones are involved in the iron-deficient signaling in strategy I plants, such as ethylene, auxin and NO, which increase significantly under iron deficiency (Romera et al., 1992; Lucena et al., 2006; Graziano and Lamattina, 2007; Chen et al., 2010; García et al., 2010, 2011; Bacaicoa et al., 2011; Lingam et al., 2011; Meiser et al., 2011; Ramírez et al., 2011). However, for plants that use strategy II, information on the signaling molecules that respond to iron deficiency is limited (Wu et al., 2011). In rice, brassinosteroids negatively regulate the iron-related genes under iron deficiency, and jasmonate pathway signaling is activated in the early stage of iron deficiency in rice roots (Wang et al., 2015; Kobayashi et al., 2016).

In this study, we found the distribution of iron caused different physiological responses to iron deficiency in different rice genotypes, including those of leaf and root ultrastructure, photosynthesis and ROS system. Furthermore, split root and shoot removal experiments were used to determine whether the regulation of iron-induced gene expression in the roots was promotive or repressive and which part of the shoot generated the signal. The expression of iron regulated marker genes were also investigated. We proposed that long-distance signals were generated in shoots associated with the regulation of iron uptake from roots, and that the signal strength was highly correlated with functional leaves.

## Materials and Methods

### Plant Materials

Two indica rice cultivars H 9405 and Yang 6 were used in this study. H 9405 is a Fe-enriched cultivar, whereas Yang 6 is a low-Fe seed accumulation cultivar (**Table 1**).

**Table 1:** Fe content distribution in Yang 6 and H 9405 (mg/kg).

	Paddy	Brown rice	Polished rice	The first complete leaf	The second complete leaf	Root
						
Yang 6	6.7	4.8	2.2	0.418	0.768	1.614^∗^
H 9405	17.3^∗∗^	7.4^∗^	10.9^∗∗^	0.93^∗∗^	1.191^∗^	1.293

*Fe content in seeds, shoot and root were measured in Fe sufficient condition. Asterisk represents significantly differences at *P* = 0.05 level. The original data has been published in our earlier study (Chen et al., 2014)*.

Seeds of the two varieties were soaked and pre-germinated for 72 h in the dark. Germinated seeds were selected and transferred to plastic containers filled with moist quartz sand. Plants were grown for 2 weeks before transplanting into 7 L plastic containers filled with half-strength Yoshida’s rice nutrient solution (Yoshida et al., 1976) for 1 week and then were grown in a complete modified Yoshida’s rice nutrient solution for another 2 weeks (Zhao et al., 2012). Complete strength nutrient solutions contained 10 mg L^-1^ NaH_2_PO_4_ •2H_2_O, 40 mg L^-1^ K_2_SO4, 40 mg L^-1^ CaCl_2_, 40 mg L^-1^ MgSO_4_ •7H2O, 0.5 mg L^-1^ MnCl_2_ •4H_2_O, 0.05 mg L^-1^ (NH_4_)_6_ •Mo_7_O_24_2H_2_O, 0.2 mg L^-1^ H_3_BO_3_, 0.01 mg L^-1^ ZnSO_4_ •7H_2_O, 0.01 mg L^-1^ CuSO_4_ •5H_2_O, and 2.0 mg L^-1^ Fe(III)-EDTA and 10 mg L^-1^ N (the concentration of N was maintained by NH_4_NO_3_). Then, rice plants were grown under Fe-deficient (0) and Fe-sufficient (2.0 mg L^-1^) conditions. The pH was adjusted to 5.0 ± 0.2 with HCl to maintain identical conditions. We refreshed the hydroponic solution every 2 days. For the samples for ROS enzymes, leaves and roots were harvested after 2 weeks of the treatments and were frozen in liquid nitrogen and stored at -70°C.

For split root experiment, 2-week-old seedlings’ roots were divided into half, and transferred to a specialized container, half of the container was filled with hydroponic solution of full strength, and the other half solution was depleted with Fe(II)-EDTA. Shoot and root tissues were harvested at day 3, 5, and 7 after the treatment and snap-frozen in liquid nitrogen before the gene expression levels were analyzed.

For the shoot removal experiment, the 2-week-old seedlings were treated with the following treatments: A, all leaves; B, all leaves removed; C, all mature leaves removed, with the fresh leaf intact; D, removed all the leaves besides of the fresh leaf; E, the upper functional leaves removed; F, the oldest leaves removed. All the excised patterns we set both Fe sufficient and Fe deficient hydroponic solution. At day 3, 5, 7 after the treatment, the roots were harvested to extract mRNA for analysis. All samples were frozen in liquid nitrogen and stored at -70°C.

### Photosynthetic Rate

The leaf photosynthetic rate (Pn) was measured with an LI-6400 (Li-Cor Inc., United States). After 2 weeks of iron deficiency, a functional leaf was used to measure the Pn.

### ROS Enzymes

The concentration of lipid peroxidation in plant tissue was determined measuring the content of malondialdehyde (MDA), which is a primary thiobarbituric acid reactive species (TBARS) and product of lipid peroxidation (Hodges et al., 1999). Tissue samples (0.1 g) were homogenized in 5 mL of TCA (0.1%, w/v) before centrifuging at 10,000 × *g* for 10 min. For measurement of MDA concentration, a 1 mL aliquot of the supernatant solution was mixed with 4 mL of 20% TCA with 0.5% TBA. The mixture was heated at 95°C for 20 min, cooled in an ice bath, and centrifuged at 10,000 × *g* for 10 min at 4°C. The absorbance of the supernatant solution was measured at 532 and 600 nm. The concentration of MDA was calculated using an MDA extinction coefficient of 155 mmol L^-1^ cm^-1^. The MDA results are expressed as mol g^-1^ FW. The H_2_O_2_ was measured according to the protocol of Patterson et al. (1984).

Superoxide dismutase (SOD) activity was assayed measuring the capacity to inhibit the photochemical reduction of NBT (Tan et al., 2008). The reaction mixture (3 mL) contained 130 mmol L^-1^ methionine, 750 mol L^-1^ NBT, 100 mol L^-1^ EDTA-Na, and 50 μL of enzyme extract in 50 mmol L^-1^ phosphate buffer (pH 7.8). To start the reaction, 20 mol L^-1^ riboflavin was added, and the cuvette was exposed to a 15-W circular “white light” tube for 10 min. The absorbance of the reaction mixture was measured at 560 nm. The amount of enzyme that inhibited 50% of the photochemical NBT reduction per fresh mass (FM) of sample was defined as the SOD activity.

Peroxidase (POD) activity was measured monitoring the change in absorption at 470 nm that resulted from guaiacol oxidation (Tan et al., 2008). A reaction solution (3 mL) that contained 0.2 mol L^-1^ PBS (pH 6.0), 29% H_2_O_2_, and guaiacol was used. The reaction was started with the addition of 10 μL of enzyme extract. Changes in absorbance were recorded at 470 nm at 1-min intervals within 3 min of the start of the reaction.

Ascorbate peroxidase (APX) activity was measured monitoring the rate of ascorbate oxidation at 290 nm (Nakano and Asada, 1981). The reaction mixture contained 0.25 mmol L^-1^ ASA, 1.0 mmol L^-1^ H_2_O_2_, 0.1 mmol L^-1^ EDTA, and 0.1 mL of enzyme extract in 50 mmol L^-1^ potassium phosphate buffer (pH 7.0). The APX activity was calculated as mol ASA mg^-1^ FW min^-1^.

Glutathione reductase (GR) activity was determined by the oxidation of NADPH measured at 340 nm (Foyer and Halliwell, 1976). A reaction mixture (1 mL) that contained 50 mmol L^-1^ potassium phosphate buffer (pH 7.8), 2 mmol L^-1^ Na2EDTA, 0.15 mmol L^-1^ NADPH, and 0.5 mmol L^-1^ GSSG was added to 225 μL of the enzyme extract. The reaction was initiated with the addition of NADPH. The absorbance of the assay mixture in the absence of NADPH at 340 nm was used for background correction.

### Total RNA Extraction and Analyses

Total RNA was extracted from rice roots using an E.Z.N.A. _ Plant RNA Kit (Omega Bio-tek, Inc., United States). The reverse transcription reaction was performed with a PrimScriptTM RT reagent Kit (Takara, Kyoto, Japan), oligo-dT, and random hexamer primers according to the manufacturer’s protocol. A quantitative real-time PCR (qRT-PCR) was performed using an ABI 7300 sequencer and SYBR Premix Ex TaqTM (Takara, Kyoto, Japan) according to the manufacturer’s protocol. The PCR and the quantitative real-time PCR primers are shown in **Table 2** (Cheng et al., 2007).

**Table 2:** Primers used for real-time PCR.

Gene	Forward primer 5′→ 3′	Reverse primer 5′→ 3′
*OsYSL15*	GGATTGCAGAAATAAACAGTGATG	TGCCAAACTAAACAATTCTCAA
*OsYSL2*	GAGGGACAACGGTGTCATTGCTGGT	TGCAGAAAAGCCCTCGACGCCAAGA
*OsNAS1*	GTCTAACAGCCGGACGATCGAAAGG	TTTCTCACTGTCATACACAGATGGC
*OsNAS2*	TGAGTGCGTGCATAGTAATCCTGGC	CAGACGGTCACAAACACCTCTTGC
*Actin*	CAATCGTGAGAAGATGACCC	GTCCATCAGGAAGCTCGTAGC

## Results

To investigate the adaptive mechanism of rice grown under iron deficiency, proteins differentially accumulated in leaves and roots of Yang 6 under Fe deficiency growth condition were profiled using a two-dimensional electrophoresis (2-DE) and Matrix-Assisted Laser Desorption/Ionization Time of Flight Mass Spectrometry (MALDI-TOF/MS) in our earlier study (Chen et al., 2015). Accumulations of 73 proteins were detected to be increased or decreased upon iron deficiency, and sixty three of them were successfully identified. Among the sixty three proteins, a total of 40 proteins were identified in rice leaves, and twenty three proteins were in roots. Most of these proteins are involved in photosynthesis, carbohydrate metabolism, oxidative stress, Adenosine triphosphate (ATP) synthesis, cell growth or signal transduction. The results provide a comprehensive way to understand, at the level of proteins, the adaptive mechanism used by rice shoots and roots under iron deficiency. Based on the discovery from the proteomic information under iron deficiency, special attention was paid to rice growth status, photosynthetic characteristics, anti-oxidation systems, marker genes correlated with Fe uptake and transport and signal transduction in this study.

### Effects of Iron Deficiency on Anti-oxidative System Within Two Rice Genotypes

**FIGURE 1:**
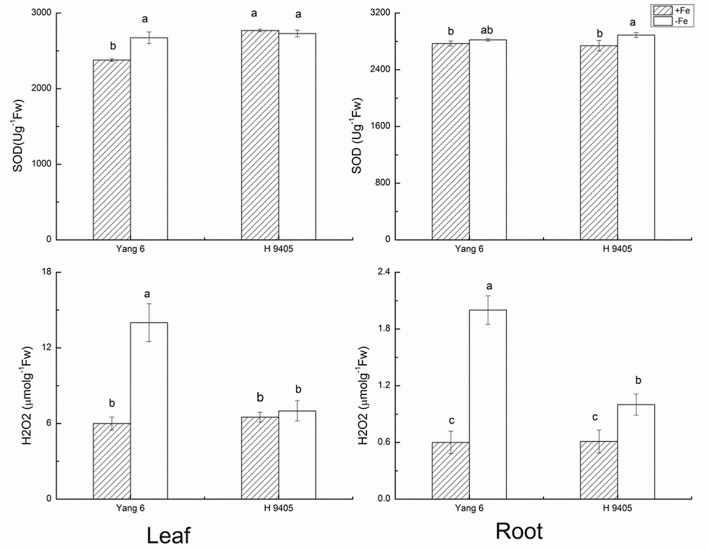
Effects of iron deficiency on SOD and H_2_O_2_ contents in leaves and roots of rice seedlings. Data are presented as means ± SE of three biological replicates. Significant differences among treatments are indicated by different letters as determined by Duncan’s test (*P* < 0.05).

Under iron deficiency, H 9405 and Yang 6 showed different response in the anti-oxidative system. We measured some marker enzymes involved in the detoxification of dangerous forms of oxygen, and regeneration of reducing agents, within the two varieties in both shoots and roots. As shown in **Figure 1**, the content of H_2_O_2_ increased in both leaf and root in the two varieties under iron deficiency. In Yang 6, the H_2_O_2_ content in leaf and root increased by 133.3 and 233.3%, respectively, compared with the control plants. A much smaller increase occurred in the roots of H 9405 than that in Yang 6, whereas leaf content of H_2_O_2_ of H 9405 did not change under Fe deficiency. The trends for the content of SOD were similar to those of H_2_O_2_ within the two varieties (**Figure 1**).

The indicator MDA represents the degree of damage to cell membranes. In Fe-deficient leaf and root of Yang 6, MDA content increased by 87.5 and 200%, respectively. In H 9405, the increase in MDA content in the root was half that of the Fe-sufficient plant, and no significant change occurred in the leaf.

**FIGURE 2:**
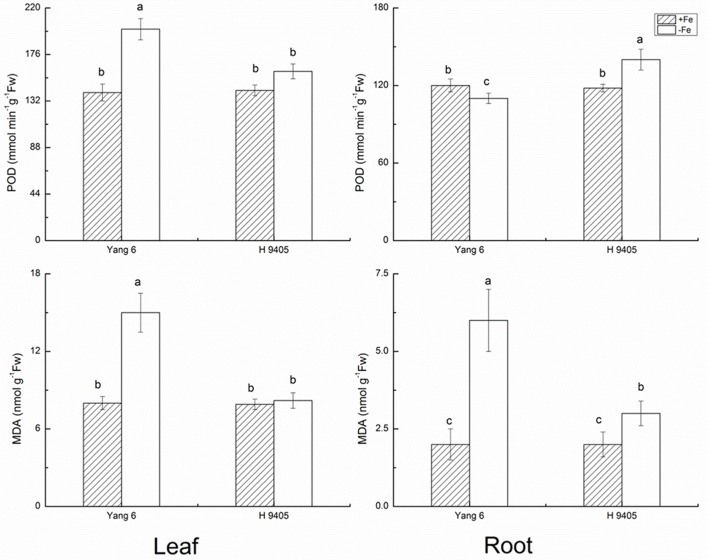
Effects of iron deficiency on POD and MDA contents in leaves and roots of rice seedlings. Data are presented as means ± SE of three biological replicates. Significant differences among treatments are indicated by different letters as determined by Duncan’s test (*P* < 0.05).

The bio-function of POD in plants includes the elimination of H_2_O_2_ under abiotic stress. However, under abiotic stress and the initial stage of senescence, POD can participate in the synthesis of radicals, chlorophyll degradation and cytol membrane damage. As shown in **Figure 2**, POD content increased by 42.9% in Yang 6 leaf under iron deficiency and by 12.7% in leaf of H 9405. In roots, no significant increase was detected in Yang 6, but POD increased 18.6% in H 9405 under iron deficiency.

**FIGURE 3:**
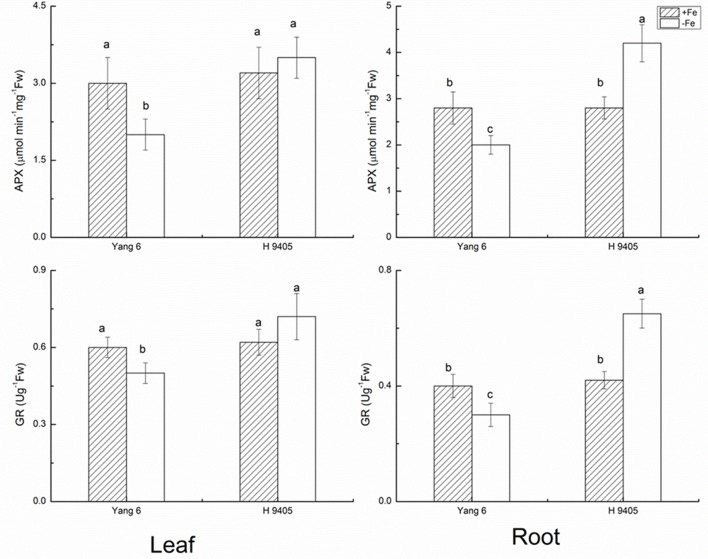
Effects of iron deficiency on APX and GR contents in leaves and roots of rice seedlings. Data are presented as means ± SE of three biological replicates. Significant differences among treatments are indicated by different letters as determined by Duncan’s test (*P* < 0.05).

APX catalyzes the conversions of H_2_O_2_ and ascorbic acid into MDHA. The content of APX decreased in leaf and root by 33.3 and 28.6% in Yang 6 under iron deficiency, whereas in H 9405, APX content increased 50% in the root compared with the control, and no significant change occurred in the leaf. The trends for GR content were similar to those for APX (**Figure 3**).

Our results showed in H 9405, the leaf’s anti-oxidative enzymes showed no changes, while in root, they were activated. In Yang 6, the detoxification cycle was activated in both shoots and roots. Therefore, compared with H 9405, Yang 6 was more sensitive and the defense system was destroyed under iron deficiency.

### Effects of Fe Deficiency on Leaf Photosynthesis, Shoot/Root Ratio and Cell Ultrastructure

**FIGURE 4:**
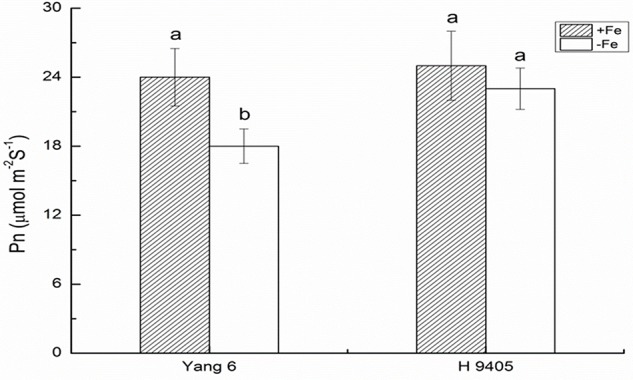
Effects of iron deficiency on the photosynthetic rate on rice of different genotypes. Data are presented as means ± SE of three biological replicates. Significant differences among treatments are indicated by different letters as determined by Duncan’s test (*P* < 0.05).

**FIGURE 5:**
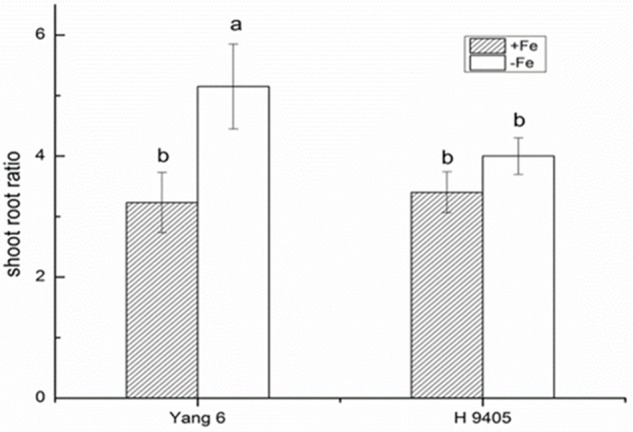
Effects of iron deficiency on shoot-root ratio of Yang 6 and H 9405. Data are presented as means ± SE of three biological replicates. Significant differences among treatments are indicated by different letters as determined by Duncan’s test (*P* < 0.05).

Iron functions in the biosynthesis of chlorophyll, and many enzymes involved in the photosynthetic system contain iron. Therefore, iron deficiency can affect the photosynthesis and respiration process. We measured the Pn, mesophyll cell and root tip cell ultrastructure. Under iron deficiency, Yang 6 showed a significant decrease by 25% in Pn while H 9405 showed no change (**Figure 4**). Our data also showed the shoot/root ratio increased under Fe deficiency in rice. In Yang 6, the ratio increased from 3.23 to 5.15, whereas the increase was not significant for H 9405 (**Figure 5**).

Also, the change in ultrastructure of mesophyll cells and root tip cells under the iron-deficient condition in the two varieties were observed by TEMs, damage to leaf and root ultrastructure under iron deficiency was much obvious in Yang 6 than in H 9405.

**FIGURE 6:**
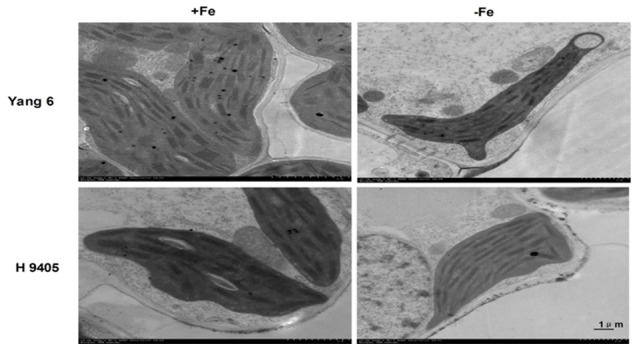
Effects of iron deficiency on mesophyll cell ultrastructure.

In Yang 6, the shape of chloroplasts and formation of granum were influenced by iron-deficiency. The chloroplast became much smaller and narrower, a circle formed by the end side. The number of starch granules decreased in iron-deficient mesophyll cells, and the thylakoid became much thinner. In H 9405, the chloroplast also became smaller, and the starch granules also decreased, but the range of change was much lighter than that in Yang 6 (**Figure 6**).

**FIGURE 7:**
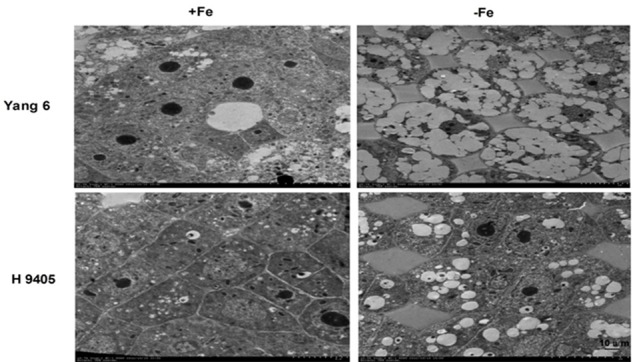
Effects of iron deficiency on root tip cell ultrastructure.

In iron-deficient rice roots, vacuoles occupied the entire cell, the nucleolus disappeared, and the membrane structure was also destroyed. The extent of damage of root ultrastructure of Yang 6 was also clearly much more severe than that of H 9405 (**Figure 7**).

### Split Root Experiment Implying the Long Distance Involvement in Fe Deficient Rice Plants

**FIGURE 8:**
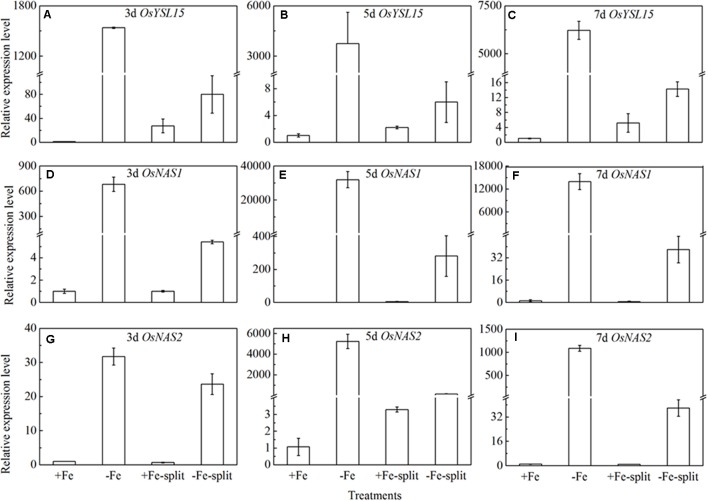
Expression levels of *OsYSL15, OsNAS1* and *OsNAS2* in rice root of the split root experiment. The part labels in figures indicate the expression levels of different genes after different days (3, 5, and 7) of iron deficiency treatment. **(A–C)** Expression levels of *OsYSL15* at 3, 5, and 7 days; **(D–F)** Expression levels of *OsNAS1* at 3, 5, and 7 days; **(G–I)** Expression levels of *OsNAS2* at 3, 5, and 7 days. Data are presented as means ± SE of three biological replicates.

**FIGURE 9:**
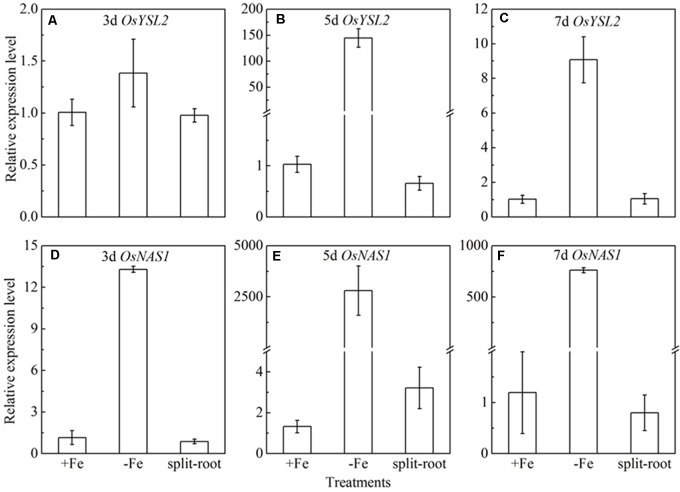
Expression levels of *OsYSL2* and *OsNAS1* in rice shoot of the split root experiment. The part labels in figures indicate the expression levels of different genes after different days (3, 5, and 7 days) of iron deficiency treatment. **(A–C)** Expression levels of *OsYSL2* at 3, 5, and 7 days; **(D–F)** Expression levels of *OsNAS1* at 3, 5, and 7 days. Data are presented as means ± SE of three biological replicates.

Split root experiment is a classic method to prove the long distance signal. We used a special container with a plastic board in the middle, half filled with full nutrition, and the other half solution without Fe, then put half root part in the nutrition with Fe, and the other half part root in the Fe deficient solution. We measured the expression levels of *OsYSL15*, *OsNAS1* and *OsNAS2* in root (**Figure 8**), and also the expression levels of *OsYSL2* and *OsNAS1* in shoot (**Figure 9**) at day 3, 5, and 7 of the treatment. In root, the expression levels of *OsYSL15*, *OsNAS1*, and *OsNAS2* were significantly induced by iron deficiency. As shown in **Figure 8**, the expression levels of the above genes in split root were both increased under iron sufficient and deficient solution in the split system, and also the induced level was much lower than that with whole root under iron deficiency. If the Fe deficient signal is local, the marker genes expression levels of iron deficient root in the split system with Fe hydroponic will not increase, but we can see a obvious increase. And also the genes expression levels in the split system without Fe showed increase, but not as much as the whole root under Fe deficiency, indicating the communication between the split root. The shoot might be the interacted organ of the rice plant. Next, we want to prove whether the shoot can generate the signal under iron deficiency.

### Shoot Removal Experiment Indicating the Long Distance Signal Is Correlated With Functional Leaf

**FIGURE 10:**
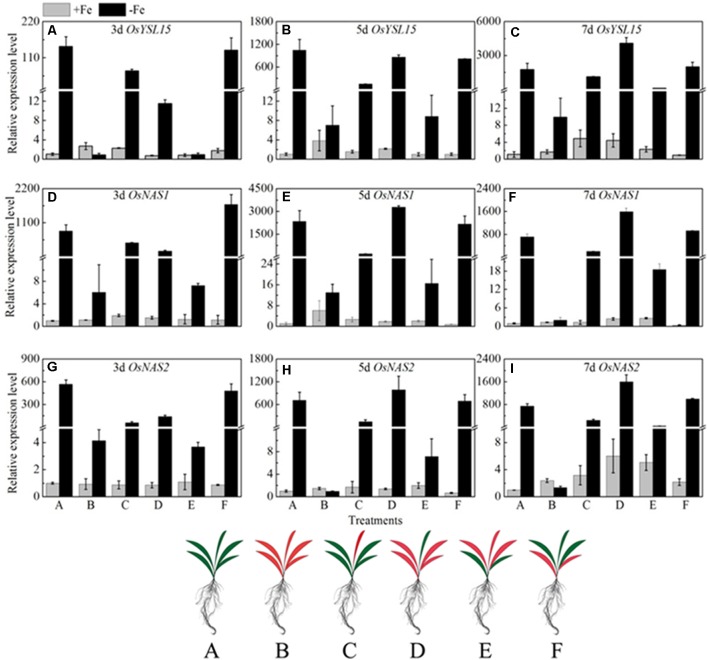
Effects of shoot excision on expression levels of *OsYSL15, OsNAS1* and *OsNAS2* in root in Yang 6. qRT-PCR analysis of genes in Yang 6 roots (*n* = 3, mean values with SD). The part labels in figures indicate the expression levels of different genes after different days of iron deficiency treatment. **(A–C)** Expression levels of *OsYSL15* at 3, 5, and 7 days; **(D–F)** Expression levels of *OsNAS1* at 3, 5, and 7 days; **(G–I)** Expression levels of *OsNAS2* at 3, 5, and 7 days. The value obtained from the control plant (A) with sufficient iron was arbitrarily set at 1.0. A, all leaves; B, all leaves removed; C, all mature leaves removed, with the fresh leaf intact; D, removed all the leaves besides of the fresh leaf; E, the upper functional leaves removed; F, the oldest leaves removed.

If the long distance signals involved in iron uptake are generated in leaves, the transmission of the signals could be stopped by removing entire leaves; consequently the expression of *OsYSL15, OsNAS1* and *OsNAS2* should be changed. Leaf excision experiments were carried out to clarify the hypothesis. We established different patterns of the shoot excised experiments: A, all leaves; B, all leaves removed; C, all mature leaves removed, with the fresh leaf intact; D, removed all the leaves besides of the fresh leaf; E, the upper functional leaves removed; F, the oldest leaves removed. Roots were harvested 3, 5, and 7 days after the shoot removal treatments and were used to analyze the expression levels of *OsYSL15, OsNAS1*, and *OsNAS2*. From **Figure 10**, in treatment B with all the leaves excision, the expression levels of the above three marker genes were pretty low in both Fe-sufficient and Fe-deficient plants, which means the decrease of expressions of iron uptake genes in rice roots might be caused by an absence of long distance signal. In order to elucidate which part of the shoot is involved in the signal generation, we measured the *OsYSL15, OsNAS1*, and *OsNAS2* after the leaves were excised at different positions. The gene expression levels in all patterns under Fe-sufficient conditions were very low. Under Fe-deficient condition, we can see high expression levels in both control plant and the F treatment (only functional leaves left); simultaneously, when all the mature leaves were removed and the fresh leaf remained, the level of expression was pretty low, which indicated that the fresh leaf was not essential in the signal transduction. From treatments E and F, we observed that the effect of functional leaves on the induction of *OsYSL15* expression level was much greater than that of the fresh leaf. Partial removal of the functional leaves weakened the expression level of *OsYSL15*. When all shoot parts were removed, no expression of *OsYSL15* was detected; thus, the shoot part had a relatively quantifiable relationship with the level of induction of *OsYSL15* expression. The same trend of the expression change in *OsNAS1 and OsNAS2* were observed.

## Discussion

In an earlier study, the genes involved in acquisition of Fe from soil in Yang 6 and H 9405 were both up-regulated in roots under Fe-deficiency, and the elevation of expression was much higher in Yang 6 than that in H9405. However, remobilization-related genes in shoot vasculature were expressed oppositely between the two varieties: in H 9405, the expression of these genes was up regulated, whereas in Yang 6, the expression was reduced. Our results in that earlier study show that the differential expression of root uptake and shoot-remobilization genes in the two cultivars is correlated with the Fe content in roots, shoots, and seeds. In brown rice and polished rice, the iron content in H 9405 is significantly higher than that in Yang 6; particularly in polished rice, the Fe content was approximately 5-fold higher. We also measured the Fe content of different organs in the two genotypes after treatment, and the Fe content in shoots of H 9405 was 1–3-fold higher than that in Yang 6, whereas in roots, the content of Fe in Yang 6 was significantly higher than that in H 9405 (Chen et al., 2014, 2015). Based on those results, we proposed that under iron deficiency, a shoot-borne signal is correlated with the iron distribution, and that the signal has an important role in inducing physiological and morphological alterations.

Based on the discovery from the rice proteomic study under iron deficiency (Chen et al., 2015), photosynthetic characteristics, anti-oxidation systems and marker genes correlated with Fe uptake and transport and signal transduction pathway played key roles. In this study, under iron deficiency, the damage to the ultrastructure of shoot and root cells was dramatic in Yang 6, whereas the degree of damage was much less in H 9405. Yang 6 also showed a significant decrease in Pn under iron deficiency, whereas the rate in H 9405 showed no change. Additionally, leaf and root ultrastructure damage under iron deficiency was much greater in Yang 6 than in H 9405. Furthermore, the shoot-to-root ratio increased under iron deficiency (**Figure 5**), which suggested that the transport of sucrose from shoot to root was damaged because of iron deficiency. More lateral roots emerged to absorb the iron surrounding the rhizosphere. For the iron content in roots, the concentration in Yang 6 was much higher than that in H 9405. Therefore, when the two cultivars simultaneously sense an iron deficiency, the response should be most severe in H 9405 because of the lower iron content in roots. However, the opposite response was observed, which could be explained by a long-distance signal originated in the shoot.

For the critical enzymes involved in the ROS system, the trends were similar to those of shoot and root ultrastructure. In H 9405, the antioxidative enzymes of the leaf showed no significant changes, whereas in roots, these enzymes were activated. In Yang 6, the detoxification cycle was activated in both shoots and roots. In shoots, the response of ROS enzymes was consistent with the degree of cell ultrastructure damage in the two varieties. In Yang 6, contents of H_2_O_2_ and MDA increased significantly, but no significant change was observed in H 9405. However, in roots, the contents of both increased under iron deficiency. Additionally, under iron deficiency, the trends of change of SOD and POD activity were similar to that of H_2_O_2_. We also measured two key enzymes that participate in the re-generation of the ROS system. In Yang 6 leaf, the GR content increased, but in the roots, the content decreased. The content of APX decreased in both leaf and root in Yang 6, which might be because iron is required in the biosynthesis of APX. In H 9405, APX content increased slightly in the root. In an earlier study, under iron deficiency, the rate of iron decrease was less in the roots of H 9405, and because the iron content might be sufficient for biosynthesis, the content of APX increased. The GR is downstream of APX in the ROS cycle and therefore showed a similar trend in the two varieties in this study. The different responses might also be due to the iron distribution in the two varieties. The iron content in the shoot of H 9405 was higher than that in Yang 6, and under iron deficiency, the antioxidative enzyme activities did not change significantly; thus, the increase in activity in Yang 6 might due to the lower iron content in the shoot. Therefore, Yang 6 was much more sensitive than H 9405 to iron deficiency in the shoot. Based on the comparison of the different responses of the two varieties under iron deficiency, we propose a signal originated in the shoot and was transmitted into the root to induce the ROS system, and because the reduced iron content in Yang 6 leaf resulted in a stronger signal, the range of increase in Yang 6 was highly significant. From the ultrastructure data and the ROS enzymes data, we can obvious observed the clues of the existence of the long distance signal involved in the iron deficient rice plants.

Recent experiments using approaches such as split-root and leaf defoliation clearly show that shoot-borne signals produced in mature leaves or the shoot apex and transmitted in the phloem have important roles in inducing physiological and morphological alterations (Landsberg, 1981; Enomoto et al., 2007). Although we propose that shoot-borne signals are involved in an integrative physiological response under Fe deficiency, the nature of signals involved in this whole-plant regulation of iron uptake remain unknown (Chen et al., 2014). Split root and shoot excision experiments were adopted in our study to study the signal originates. We found removal of leaves affected the expression level of the marker gene in roots under iron deficiency in a quantitative relationship between the part removed and the range of induction of the marker gene. In the treatment with all leaves removed, *OsYSL15, OsNAS1, and OsNAS2* were not induced under iron deficiency. In tobacco leaf excision experiments, the root tissue in which the iron uptake genes express doesn’t lose bioactivity by leaf removal (Enomoto et al., 2007). Similarly, a single new fresh leaf did not induce the marker gene under iron deficiency; however, the mature leaves did induce the marker gene, and the signal intensity was in proportion to the amount of mature leaves. All the above data indicate a long-distance signal occurred in iron-deficient rice plants with the signal intensity related to the mature leaves. In a future experiment, we will focus on identification of phloem signals.

**FIGURE 11:**
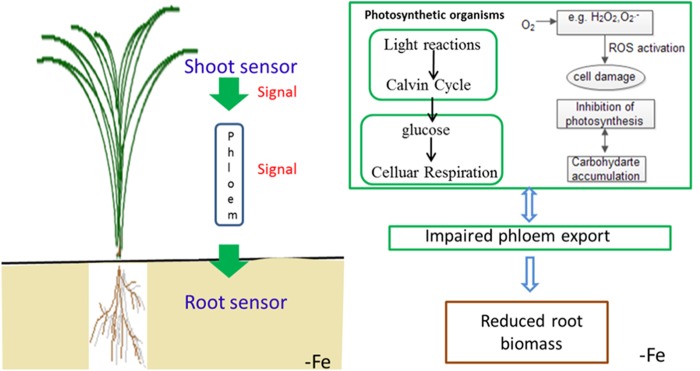
Schematic presentation of changes in signal transduction, photo-oxidative damage and carbohydrate transport in rice under iron deficiency.

To summarize the above discussion, with less iron in the shoot, Yang 6 might generate a stronger signal in the shoot than that of H 9405; thus, Yang 6 was more sensitive to iron deficiency. A reasonable conclusion is that rice differs in the response to iron deficiency because of the shoot-root iron distribution, and that the physiological alterations are at least partially controlled by the signals generated in the shoots but transmitted to roots via phloem. When rice encounters iron deficiency, the photosynthetic system will be destroyed, the ROS system will be activated, and carbohydrates will be accumulated in the shoot, with impaired phloem export increasing the shoot/root ratio (**Figure 11**).

## Author Contributions

LC and YD contributed equally to this work. LC, GW, PC, and HZ conducted the physiological experiments. SW and YD supervised the experiments. LC and YD wrote the manuscript.

## Conflict of Interest Statement

The authors declare that the research was conducted in the absence of any commercial or financial relationships that could be construed as a potential conflict of interest. The handling Editor and the reviewer D-YC declared their shared affiliation.
